# Analysis of the association between adiponectin, adiponectin receptor 1 and diabetic cardiomyopathy

**DOI:** 10.3892/etm.2014.1539

**Published:** 2014-02-12

**Authors:** JIEHUA LI, SHIYUE SU, XIAONA ZONG

**Affiliations:** Department of Geriatric Medicine, The First Affiliated Hospital of Anhui Medical University, Hefei, Anhui 230022, P.R. China

**Keywords:** diabetic cardiomyopathy, adiponectin, adiponectin receptor

## Abstract

The aim of this study was to explore the association between adiponectin (APN), APN receptors and insulin resistance (IR) using rats with type 2 diabetes mellitus (T2DM) as a model of diabetic cardiomyopathy (DC). Serum and cardiac APN levels were assessed using a double-antibody sandwich ELISA. In addition, the mRNA and protein expression of the myocardial APN receptor 1 (AdipoR1) was determined using the reverse transcription polymerase chain reaction and immunohistochemical staining. The results showed that the heart weight/body weight ratio, fasting plasma glucose (FPG) and lipid levels, and the homeostasis model assessment-estimated IR (HOMA-IR) index were elevated in the T2DM group compared with the control group. Cardiac function was significantly lower in the T2DM group compared with the control group (P<0.05). Furthermore, serum and cardiac APN levels were significantly reduced in the T2DM group compared with the control group, and mRNA and protein expression of AdipoR1 was lower in the T2DM group compared with the control group (P<0.05). Changes in the morphology of myocardial cells were observed under the light microscope using hematoxylin and eosin staining. Myocardial cell hypertrophy, a disordered cell arrangement and irregular nuclear size were observed in the T2DM group. By contrast, myocardial cells in the control group were arranged in neat rows with uniform cytoplasmic and nuclear staining. According to the correlation analyses, serum APN levels in the T2DM group were negatively correlated with FPG, triglyceride, total cholesterol and fasting insulin (FINS) levels, as well as with the HOMA-IR index. Myocardial AdipoR1 protein expression was positively correlated with myocardial APN levels, and negatively correlated with FINS and HOMA-IR. It may be concluded that myocardial and serum levels of APN are reduced in rats with DC. Metabolic disorders of blood glucose and lipid levels, as well as IR, are associated with low APN levels. Furthermore, low levels of myocardial Adipo1R mRNA and protein expression correlate with reduced insulin sensitivity.

## Introduction

The etiology of diabetic cardiomyopathy (DC) can not be explained by hypertensive, coronary or valvular heart disease, or other cardiac lesions. There is a lack of consensus regarding the pathogenesis and diagnosis of DC, and a standard treatment has yet to be established. Factors that are recognized to be involved in the pathogenesis of DC include metabolic disorders, myocardial fibrosis, microvascular disease, autonomic disorders and insulin resistance (IR) (1). Among these factors, IR is the main indicator across the various metabolic diseases. Furthermore, IR is involved in the pathophysiology of diabetes, hypertension, hyperlipidemia and other metabolic diseases. IR is also one of the most important factors to promote the development of DC.

Adiponectin (APN), a fat cell factor secreted by myocardial cells, regulates cardiac function and myocardial metabolism through autocrine and paracrine signaling. The effects of APN depend on its interaction with the APN receptor (2). The binding of APN to the APN receptor, which is mediated by a series of biological mechanisms, induces anti-inflammatory, antidiabetic and antiatherogenic properties. In addition, it regulates glucolipid metabolism, increasing fatty acid oxidation and glucose uptake and enhancing insulin sensitivity (3). However, the association between APN and DC, and the importance of APN in the development of IR in patients with DC has yet to be elucidated.

The purpose of this study was to observe the changes in serum and cardiac levels of APN in a rat model of DC. Furthermore, the mRNA and protein expression of the cardiac APN receptor 1 (AdipoR1), as well as the association between APN, AdipoR1 and IR, were investigated. The results may provide a theoretical basis for the clinical use of either APN or drugs targeted to the APN pathway to reduce IR and treat DC.

## Materials and methods

### Experimental animals

Male Sprague Dawley rats (weight, 200–220 g; age, 4–6 weeks; n=61) were obtained from the Experimental Animal Center of Anhui Medical University (Hefei, China). The rats were housed with five to six rats per cage in the animal laboratory of Anhui Medical University under the following conditions: Room temperature, 18–22°C; relative humidity, 30–70%; light/dark, 12/12 h; free access to water and food. After one week of feeding, the rats were randomly divided into type 2 diabetes mellitus (T2DM) (n=40) and control (n=20) groups. The control group was fed a standard diet (64% carbohydrate, 23% protein and 13% fat) and the T2DM group was fed a high-fat, high-sugar diet (59% routine feed, 18% lard, 20% sucrose and 3% egg yolk powder). After four weeks, the T2DM group received an intraperitoneal injection of streptozotocin (STZ; 30 mg/kg), while the control group received an intraperitoneal injection of an equal volume of citrate buffer. Assessment was performed during week 14. One day prior to assessment, the rats underwent a 12–14-h fast. Body weights (BWs) were recorded and the rats were anesthetized with 10% chloral hydrate (0.3 ml/100 g BW). The anesthetized rats were placed on the operating table, tail blood glucose levels were measured with a glucose meter (Roche Diagnostics GmbH, Mannheim, Germany) and blood samples were collected.

### Assessment of cardiac function

An arterial catheter was inserted into the common carotid artery of each rat. The maximum rate of left intraventricular isovolumic systolic pressure increase (+dp/dt_max_) and decrease (−dp/dt_max_) was measured. Biological signals were collected and analyzed with an electronic BL-410 biological and functional experimental system (Chengdu Taimeng Science And Technology Co., Ltd., Chengdu, China).

### Analysis of blood lipid and insulin levels and insulin sensitivity

Aortic blood samples were collected, centrifuged at 2,800 × g for 10 min at 4°C, and the supernatants were preserved at −70°C. Triglyceride (TG) levels were measured using the glycerol-3-phosphate oxidase-peroxidase method (BoAoSen BioCompany, Beijing, China), total cholesterol (TC) levels were determined by an enzyme-linked colorimetric assay and insulin levels were measured with a radioimmunoassay (YuanZi Science Biocompany, Beijing, China). Calculations were performed to determine the insulin sensitivity index (ISI, expressed in its natural logarithm) and the homeostasis model assessment-estimated IR (HOMA-IR) index as follows: ISI=1/[fasting plasma glucose (FPG) × fasting insulin (FINS)]; HOMA-IR index=FPG × FINS/22.5.

### Specimen collection

The hearts were separated from the heart artery roots, rinsed with physiological saline, weighed and blotted with filter paper. The heart blood vessels and connective tissue were removed and the left apex was dissected in an ice bath into pieces weighing 100 mg each. Subsequently, the specimens were wrapped in tin foil, frozen with liquid nitrogen and stored at −70°C in preparation for the analysis of myocardial APN and AdipoR1 levels.

A sample of the left ventricular tissue was fixed in 4% paraformaldehyde at 4°C for 48 h. Paraffin-embedded 4-μm tissue sections were then prepared for immunohistochemical analysis and the pathological observation of the immunostained tissues using hematoxylin and eosin staining.

### Assessment of serum and myocardial levels of APN

APN levels were measured using a double-antibody sandwich ELISA (Beijing Boaosen Biotechnology Co. Ltd., Beijing, China). The sample protein concentration was calculated by recording the absorbance as optical density (OD) at a wavelength of 450 nm.

### Assessment of cardiac AdipoR1 protein expression

A streptavidin-biotin-peroxidase complex kit (Zymed Laboratories, Inc., South San Francisco, CA, USA) and anti-AdipoR1 antibody (Beijing Boaosen Biotechnology Co. Ltd.) were used to monitor the expression of cardiac AdipoR1. Semi-quantitative analyses were performed based on the area and intensity of staining using an MIAS-2000 color image processing system (Micro image analysis system-2000; Zhisheng Software Company, Chengdu, China). In each sample, five high-power fields (magnification, ×400) were randomly selected and the mean OD value was calculated.

### Expression of myocardial AdipoR1 mRNA

Levels of myocardial AdipoR1 mRNA expression were assessed with the reverse transcription polymerase chain reaction (RT-PCR). Primer sequences for AdipoR1 were: 5′-AACTGGACTATFCAGGGA-3′ (upstream primer) and 5-′TGGTPCCAGTCTCATCAG-3′ (downstream primer). The amplification fragment length was 398 base pairs. Primer sequences for the control, GAPDH, were: 5′-ATGGTGAAGGTCGGTGTG-3′ (upstream primer) and 5′-AACTTGCCGTGGGTAGAG-3′ (downstream primer). The amplification fragment length was 161 base pairs. Results of the electrophoresis gels were analyzed with an imaging system (Biosens Gel 750; Shanghai Shanfu Scientific Instrument Company, Shanghai, China). The relative content of AdipoR1 mRNA was represented by the ratio of the OD of the AdipoR1 band and the GAPDH band.

### Statistical analysis

SPSS 13.0 statistical software (SPSS, Inc., Chicago, IL, USA) was used to analyze the data. A t-test was used to compare the normally distributed data in the two groups. Data are expressed as the mean ± standard deviation. HOMA-IR indices showed a skewed distribution. Pearson’s coefficient was used for two-variable correlation analysis. P<0.05 was considered to indicate a statistically significant difference.

## Results

### Biochemical indices

FPG, TG, TC and FINS levels, and the HOMA-IR index were significantly higher in the T2DM group compared with the control group (all P<0.05) (Table I).

### BW, heart weight (HW) and cardiac function

Prior to STZ injection, the BWs of rats in the T2DM group were not significantly different from those of the rats in the control group. However, polyphagia, polydipsia, polyuria and gradual weight gain were observed in the T2DM group following STZ injection. Significant differences in BW were identified between the two groups (P<0.05). In addition, there were significant differences in HW and −dp/dt_max_ between the T2DM and the control group (both P<0.05) (Table II).

### Morphological changes in the myocardia of rats

**Figure 1:**
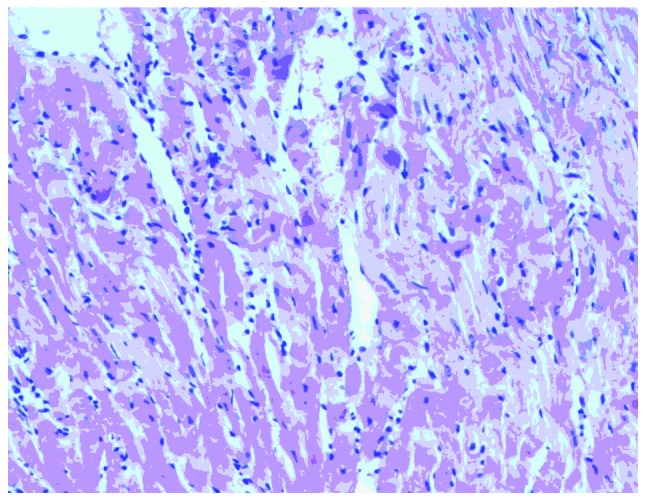
Hematoxylin and eosin staining of myocardial cells in the type 2 diabetes mellitus group. Magnification, ×400.

**Figure 2:**
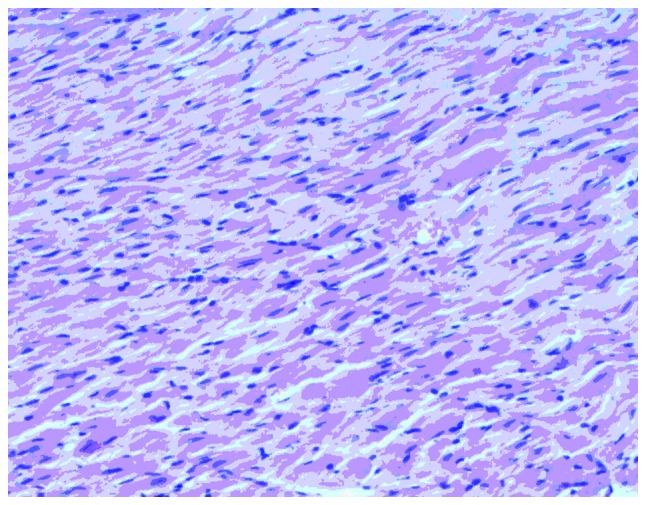
Hematoxylin and eosin staining of myocardial cells in the control group. Magnification, ×400.

Myocardial hypertrophy, cytoplasm was loose, reticulated and translucent, a disordered cell arrangement and nuclear size irregularities were observed in T2DM group rats under the light microscope. In the control group, myocardial cells were arranged in neat rows, the size of nuclei was consistent and cytoplasmic staining was uniform (Figs. 1 and 2).

### Expression of serum and myocardial APN and myocardial AdipoR1

**Figure 3:**
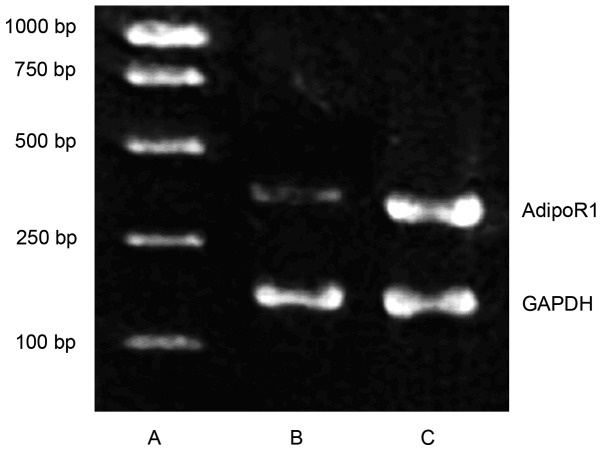
mRNA expression of myocardial AdipoR1 in the T2DM and control groups. (A) Marker; (B) T2DM group; (C) control group. AdipoR1, adiponectin receptor 1; T2DM, type 2 diabetes mellitus.

**Figure 4:**
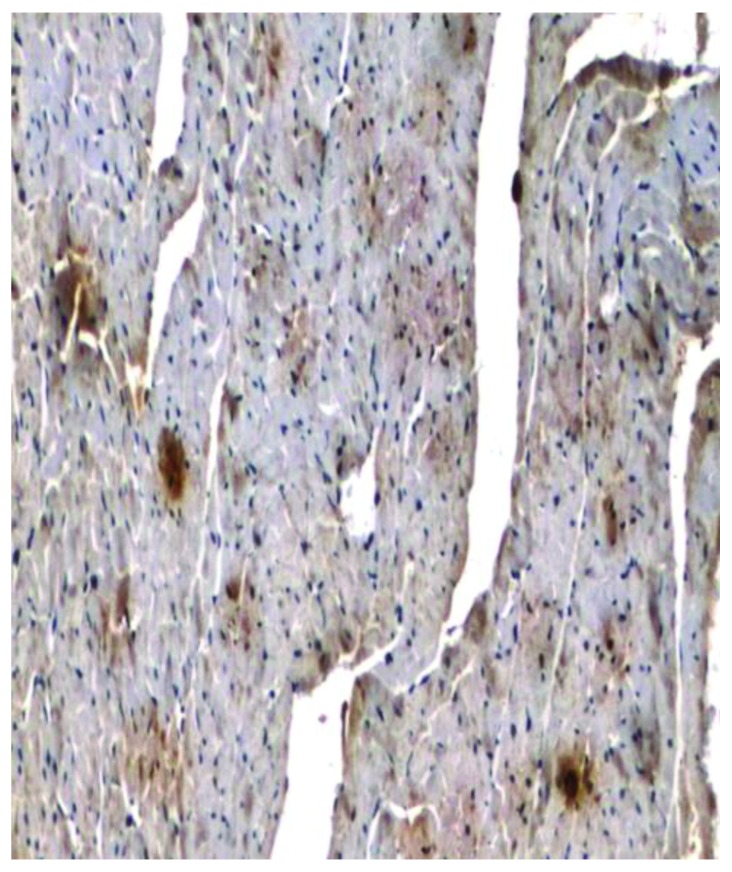
Immunohistochemical staining of myocardial adiponectin receptor 1 in the type 2 diabetes mellitus group (magnification, ×400).

**Figure 5:**
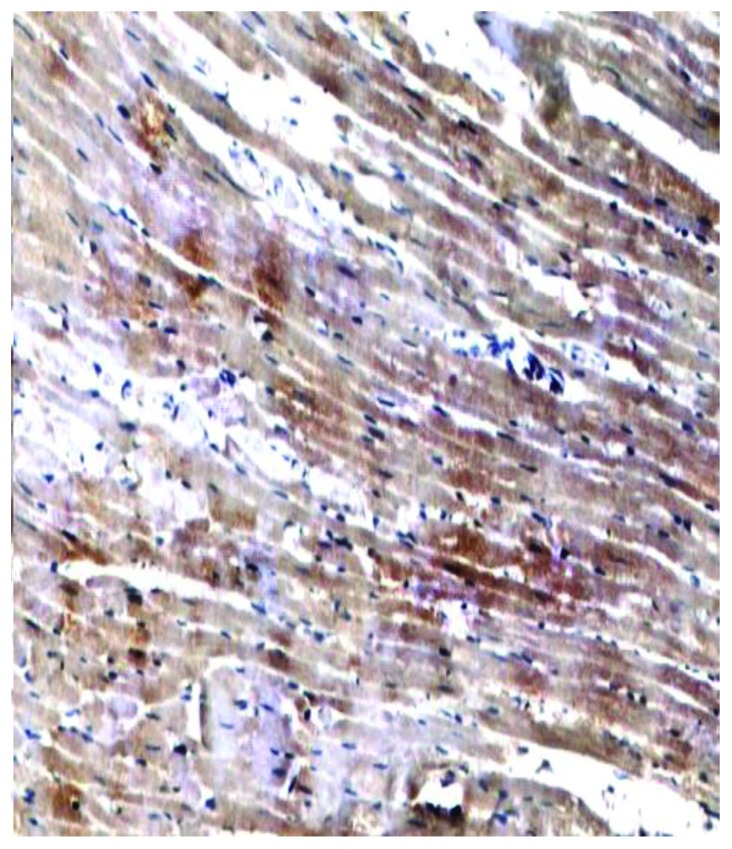
Immunohistochemical staining of myocardial adiponectin receptor 1 in the control group (magnification, ×400).

Levels of serum APN and myocardial APN were significantly lower in the T2DM group compared with the control group (both P<0.05). RT-PCR showed that AdipoR1 mRNA expression was significantly reduced in the T2DM group compared with the control group (P<0.05). Furthermore, immunohistochemical staining showed that the AdipoR1 protein was mainly located in the cytoplasm. The expression of myocardial AdipoR1 protein was significantly lower in the T2DM group compared with the control group (Table III, Figs. 3–5).

### Correlation analyses of serum APN and FPG, TG, TC, FINS and HOMA-IR in rats with T2DM

Correlation analyses of serum APN and FPG, TG, TC, FINS and HOMA-IR in the T2DM group showed that serum APN levels were negatively correlated with FPG, TG, TC, FINS and HOMA-IR (r=−0.721, −0.582, −0.549, −0.613 and −0.637, respectively; all P<0.05) (Table IV).

### Correlation analyses of AdipoR1 protein expression and myocardial APN, FINS and HOMA-IR in rats with T2DM

Correlation analyses were conducted on myocardial AdipoR1 protein expression and myocardial APN, FINS and HOMA-IR in rats with T2DM. Expression of the AdipoR1 protein was positively correlated with myocardial APN (r=0.890, P<0.05) and negatively correlated with FINS and HOMA-IR (r=−0.697, and −0.593, respectively; both P<0.05) (Table V).

## Discussion

The pathogenesis of DC has yet to be fully elucidated. The etiology of the disease includes metabolic dysfunction, IR, autonomic nervous system disorders, and myocardial fiber necrosis and apoptosis (1,4). Early symptoms of DC are not obvious and mainly manifest as ventricular diastolic dysfunction. However, aggravation of DC eventually leads to heart failure, malignant arrhythmia and sudden mortality (5).

APN, which is secreted by adipose cells, circulates in the blood and organs. Yamauchi *et al* (6) were the first group to clone human and mouse AdipoR1 and AdipoR2 cDNAs. APN and its receptor have been found to affect metabolic regulation and improve IR and oxidative stress (7). Furthermore, the function of APN in patients with diabetes mellitus has received increasing focus (7). Further study of APN and its receptors may provide novel strategies for the prevention and treatment of DC.

In the present study, FPG and FINS levels were elevated in the rats with T2DM compared with the control group, while serum APN levels were significantly decreased. APN correlation analyses revealed a strong correlation between serum APN and FPG levels. This study showed that the HOMA-IR index increased significantly in the T2DM group relative to the control group, and was negatively correlated with serum APN levels. Therefore, it may be concluded that decreased APN and abnormal glucose metabolism are interrelated, and have an important role in glucose metabolism dysfunction in patients with DC. Furthermore, IR may lead to reduced secretion of APN; therefore, increased insulin sensitivity may improve APN secretion, which is expected to become a therapeutic target for the prevention and treatment of DC.

Aberrant lipid metabolism is one of the manifestations of DC. APN is independently associated with TG, TC and low-density lipoprotein cholesterol levels. In healthy, non-diabetic individuals, APN was negatively correlated with TG, TC and low-density lipoprotein cholesterol levels (8). The present study showed that TG and TC levels were higher in the T2DM group than the control group. Correlation analyses showed that APN was negatively correlated with TG and TC levels, suggesting that APN levels and lipid metabolism are closely associated with one another. Therefore, decreased APN levels represent one cause of dyslipidemia in DC.

The present study suggests that the decline in APN levels was directly or indirectly involved in the occurrence of IR in rats with DC, and that changes in AdipoR1 expression also contribute to IR. The results showed that expression levels of myocardial AdipoR1 mRNA and protein in rats with T2DM were significantly lower than those in the control group. Therefore, reduced AdipoR1 expression is involved in IR and represents one of the mechanisms of IR in DC. In addition, protein levels of AdipoR1 were positively correlated with myocardial APN, which suggests that APN regulates the expression of the APN receptor gene.

Based on these results, the causes of IR in DC may be a decrease in APN levels, which leads to decreased binding of APN to AdipoR1. Reduced APN signal transmission subsequently downregulates the sensitivity of insulin and causes IR. The reduced expression of AdipoR1 in heart muscle affects the binding of APN to AdipoR1, thereby influencing the role of APN in regulating glucose metabolism, which leads to IR.

At present, the mechanism of APN receptor activation is not fully understood. Elucidation of the specific sites at which AdipoR1 is activated is likely to contribute to an enhanced understanding of the mechanism underlying the action of APN.

## Figures and Tables

**Table I:** Biochemical indices in the control and T2DM groups.

Group	n	FPG (mmol/l)	TG (mmol/l)	TC (mmol/l)	FINS (mU/l)	HOMA-IR (ln) (mmol/mIU/l^2^)
Control	20	5.20±0.67	0.69±0.04	1.34±0.09	11.45±1.06	0.48±0.07
T2DM	32	17.60±2.45^a^	1.67±0.11^a^	3.15±0.14^a^	20.70±1.12^a^	1.32±0.02^a^

a P<0.05 compared with the control group.

T2DM, type 2 diabetes mellitus; FPG, fasting plasma glucose; TG, triglycerides; TC, total cholesterol; FINS, fasting insulin; HOMA-IR, homeostasis model assessment-estimated insulin resistance; ln, natural logarithm.

**Table II:** BW, HW, HW/BW, −dp/dt_max_ and +dp/dt_max_ in the control and T2DM groups.

Group	n	BW (g)	HW (g)	HW/BW (g/kg)	−dp/dt_max_	+dp/dt_max_
Control	20	508.4±34.7	0.89±0.05	1.74±0.06	6037±296	7430±215
T2DM	32	427.8±24.6^a^	1.31±0.12^a^	3.10±0.11^a^	4178±129^a^	7149±189

a P<0.05 compared with the control group.

T2DM, type 2 diabetes mellitus; BW, body weight; HW, heart weight; −dp/dt_max_, the maximum rate of decrease in left intraventricular isovolumic systolic pressure; +dp/dt_max_, the maximum rate of increase in left intraventricular isovolumic systolic pressure.

**Table III:** Serum APN, myocardial APN and AdipoR1 expression in the control and T2DM groups.

Group	n	Serum APN (μg/ml)	Myocardial APN (μg/mg)	AdipoR1/GAPDH	AdipoR1 protein (OD)
Control	20	1.94±0.14	0.23±0.03	0.70±0.10	1274.19±34.86
T2DM	32	1.09±0.05^a^	0.12±0.01^a^	0.39±0.02^a^	679.44±15.13^a^

a P<0.05 compared with the control group.

APN, adiponectin; AdipoR1, adiponectin receptor 1; T2DM, type 2 diabetes mellitus; OD, optical density.

**Table IV:** Correlation analyses between serum APN and FPG, TG, TC, FINS and HOMA-IR in the T2DM group.

Variable	FPG	TG	TC	FINS	HOMA-IR
Serum APN (r-values)	−0.721^a^	−0.582^a^	−0.549^a^	−0.613^a^	−0.637^a^

a P<0.05.

T2DM, type 2 diabetes mellitus; FPG, fasting plasma glucose; TG, triglycerides; TC, total cholesterol; FINS, fasting insulin; HOMA-IR, homeostasis model assessment-estimated insulin resistance.

**Table V:** Correlation analyses between AdipoR1 protein and myocardial APN, FINS and HOMA-IR in the T2DM group.

Variable	Myocardial APN	FINS	HOMA-IR
AdipoR1 protein (r-values)	0.890^a^	−0.697^a^	−0.593^a^

a P<0.05.

APN, adiponectin; AdipoR1, adiponectin receptor 1; T2DM, type 2 diabetes mellitus; FINS, fasting insulin; HOMA-IR, homeostasis model assessment-estimated insulin resistance.
